# Inhibition of phosphodiesterase10A attenuates morphine-induced conditioned place preference

**DOI:** 10.1186/s13041-014-0070-1

**Published:** 2014-09-25

**Authors:** Ying Mu, Zhaoxiang Ren, Jia Jia, Bo Gao, Longtai Zheng, Guanghui Wang, Eitan Friedman, Xuechu Zhen

**Affiliations:** Jiangsu Key laboratory for Translational Research and Therapy for Neuropsycho-disoders & Department of Pharmacology, College of Pharmaceutical Sciences, Soochow University, 199 Ren’ai Road, Suzhou, 215123 Jiangsu Province China; Department of Physiology, Pharmacology & Neuroscience, CUNY Medical School, New York, NY USA

**Keywords:** Conditional place preference, Morphine, Nucleus accumbens, Phosphodiesterase10A, Striatum, cAMP response element binding protein, Delta FosB

## Abstract

**Background:**

Phosphodiesterase (PDE) 10A is selectively expressed in medium spiny neurons of the striatum. Nucleus accumbens (NAc) is a key region that mediates drug reward and addiction-related behaviors. To investigate the potential role of PDE10A in the reinforcement properties of morphine, we tested the effect of MP-10, a selective inhibitor of PDE10A, on acquisition, expression, and extinction of morphine-induced conditioned place preference (CPP).

**Results:**

The results show that 2.5 mg/kg MP-10, administered subcutaneously, significantly inhibited the acquisition of morphine-induced CPP. The same dose of MP-10 alone did not result in the CPP. Moreover, MP-10 did not alter the expression of morphine-induced CPP, but did accelerate the extinction of morphine-induced CPP. Additionally, chronic treatment with 2.5 mg/kg MP-10 decreased expression of phosphorylated CREB (pCREB), activated cAMP response element binding protein, in dorsomedial striatum, in shell of NAc, and in anterior cingulate cortex (ACC) as well as decreased expression of ΔFosB in the shell of NAc and ACC.

**Conclusion:**

The results suggest that inhibition of PDE10A may have therapeutic potential in the treatment of opioid addiction.

## Background

Drug addiction can be considered a chronic, recurrent brain disease. The conditioned place preference (CPP) paradigm has been widely used to study the conditioned rewarding effects of addictive drugs [1,2]. In this paradigm, the conditioned rewarding properties of drugs are evaluated by pairing drug effects with initially neutral cues, such as the compartment of an apparatus. After continuous medication, animals will display the conditioned place preference to the drug-related place [3]. The acquisition, expression and extinction of CPP provide a model that is important not only for investigating the mechanism of addiction, but also for discovering novel therapeutic approaches to addiction [1,4-6].

Cyclic nucleotides such as cyclic adenosine monophosphate (cAMP), serve as prominent second messengers in regulating a number of down-stream signaling molecules and play a critical role in a variety of cell functions. Phosphodiesterases (PDEs), which have been classified into an enzymes family consisting of 11 isozymes that hydrolyze cAMP and/or cGMP, and are essential modulators in the regulation of cAMP content in cells [7]. Among the PDE subtypes, the 10A isozyme, is a dual-substrate PDE, which is selectively expressed in medium spiny neurons (MSNs) of the striatum [8]. MSNs are striatal output neurons that represent 90% of all striatal neurons [9]. Modulation of PDE10A activity has been shown to elicit behavioral responses in experimental animals. For instance, the PDE10A inhibitor, papaverine, was found to suppress conditioned avoidance responses in rats, suggesting potential therapeutic roles in schizophrenia and in Alzhemier’s disease [10]. MP-10, 2-[4-(1-methyl-4-pyridin-4-yl-1H-pyrazol-3-yl)-phenoxymethyl]-quinoline, an analog of papaverine with excellent potency (IC50 = 1.26 nM) and selectivity for PDE10A, was found to dose-dependently increase striatal cAMP and cGMP levels in CF-1 mice, and to improve negative symptoms and cognitive function in schizophrenia-like animal models [11].

On the other hand, the ventral striatum/nucleus accumbens (NAc) is the principal region which is known to mediate drug reward and addiction-related behaviors. This brain region receives dopaminergic innervation from the ventral tegmental area (VTA) in the midbrain and is known as the mesolimbic dopamine system [12,13]. Most drugs of abuse including morphine enhance dopaminergic transmission from the VTA to the NAc and to other target limbic regions such as prefrontal cortex [14-17]. It has been previously reported that the application of a PDE4 inhibitor attenuates the rewarding properties of cocaine and morphine [18]. Given the fact that PDE10A is specifically located in striatum, an important structure involved in the reward circuit, we hypothesized the PDE10A inhibitors such as MP-10 may modulate the behavioral reinforcement exerted by morphine.

Chronic exposure to drugs of abuse will give rise to persistent structural and functional changes in the central nervous system. These phenomena are usually referred as ‘drug-induced neuroplasticity’ and depend on changes in gene expression [19]. The cAMP response element binding protein (CREB), as a downstream molecule in mediating the actions of cAMP and which MP-10 targets too, is an important transcriptional factor in establishing and maintaining addiction to drugs of abuse [13,20]. Psychostimulants increase CREB activity, as measured by increased phospho-CREB (pCREB) in multiple brain regions, including the NAc and dorsal striatum [21,22]. Phosphorylation of CREB at Ser133 activates a number of immediate early genes (IEGs) including *c-fos*. Chronic administration of drugs of abuse induces long-lasting expression of ΔFosB particularly in NAc, dorsal striatum, and prefrontal cortex; these changes persist after cessation of drug treatment [23,24]. The increased expression of ΔFosB was found to be associated with enhanced locomotion and with the rewarding effects of opiates [25,26]. In the present study we therefore also examined how modulation of PDE10A alters the expression of ΔFosB and pCREB in NAc (core and shell), dorsomedial striatum (DMS), and anterior cingulate cortex (ACC) and their relation to morphine acquisition, expression and extinction in the CPP model.

## Results

### MP-10 suppressed the acquisition but not the expression of morphine CPP

**Figure 1:**
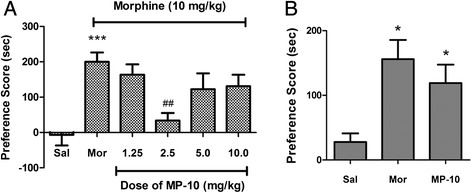
**MP-10 suppressed the acquisition (A) but not the expression (B) of morphine CPP. (A)** MP-10 (1.25-10 mg/kg, s.c.) or vehicle (2 ml/kg, s.c.) were administered 30 min before morphine (10 mg/kg, s.c.) or saline (2 ml/kg, s.c.) during the conditioning sessions. ***P < 0.001 indicates difference in comparison with saline group, whereas ##P < 0.01 indicates comparison with the morphine group. N = 6-13 per group. **(B)** Pretreatment of MP-10 (2.5 mg/kg) 30 min prior to placement in the apparatus on post-conditioning test day. *P < 0.05, compare with saline group, N = 6-10 per group. CPP scores were assessed as the difference of time spent in the drug-paired compartment between the post and pre-conditioning phases. Data were expressed as mean ± SEM.

We first tested if MP-10 suppressed the acquisition of morphine CPP. As shown in Figure 1A, after chronic administration of morphine, animals spent significantly more time in the drug-paired compartment (F_(5, 53)_ = 7.90, P < 0.0001). Significant inhibition of morphine-induced CPP was observed at 2.5 mg/kg MP-10 (t = 5.457, P < 0.001 compared with morphine group).

We also tested if administration of MP-10 alters the expression of established morphine CPP. As shown in Figure 1B, acute injection of MP-10 (2.5 mg/kg) 30 min prior to placement into the apparatus produced no significant attenuation of the previously established expression of morphine CPP.

### Chronic low dose MP-10 treatment did not produce CPP

**Figure 2:**
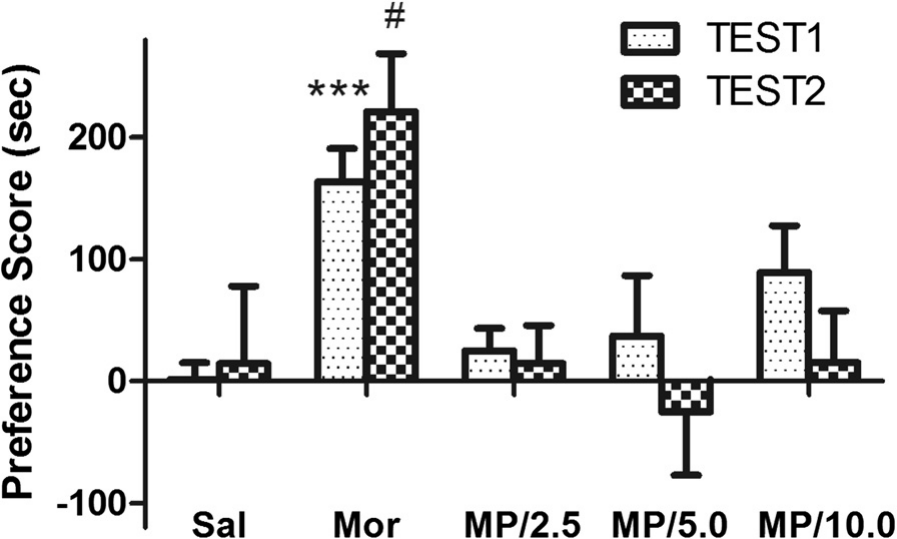
**Chronic MP-10 alone did not produce CPP.** TEST1 refers to the test after conditioning sessions, while TEST2 refers to testing one week after TEST1. During the conditioning sessions, rats in treatment group received MP-10 (2.5 -10.0 mg/kg, s.c.) instead of morphine to test for development of CPP. CPP scores were assessed as the difference in time spent in the drug-paired compartment between the post and pre-conditioning phases. Data are expressed as mean ± SEM. ***P < 0.001, compared with saline group in TEST1. #P < 0.05, when compared with saline group in TEST2. N = 7-12 per group.

In order to elucidate why high doses of MP-10 failed to suppress morphine CPP, we investigated if MP-10 alone could lead to CPP. As 2.5 mg/kg of MP-10 did not produce CPP in our preliminary experiment, we thus omitted 1.25 mg/kg group. As shown in Figure 2, after 8 days of conditioning sessions, administration of morphine caused a significant preference for the drug-paired compartment (F_(4,42)_ = 4.941, P <0.01), whereas MP-10 (2.5 mg/kg and 5.0 mg/kg, s.c.) elicited no significant CPP or aversion. At the dose of 10 mg/kg MP-10 induced a marginal CPP preference score. This may contribute to the finding that at this high dose of MP-10 the drug did not suppress morphine CPP. The apparent CPP preference score obtained with the high dose MP-10 completely disappeared when the test was re-run one week following the first test. Thus, it appears that at the high dose of MP-10 the drug produces a weak and temporary preference. However, this dose also produced catalepsy (descent latency: 47 ± 8.5 sec for 2.5 mg/kg and 116 ± 15.8 sec for 10.0 mg/kg). We thus used 2.5 mg/kg of MP-10 for the extinction experiments.

### MP-10 treatment facilitates the extinction of morphine CPP

**Figure 3:**
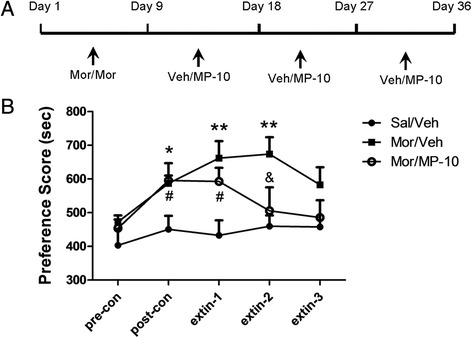
**MP-10 treatment (2.5 mg/kg, s.c.) facilitates the extinction of morphine CPP.** Figure **(A)** shows the treatment time line and figure **(B)** shows the results. No compartment preferences developed in rats receiving saline throughout the experimental procedure (Sal/Veh). Rats administered vehicle in extinction sessions show spontaneous extinction tendency until the third extinction test (Mor/Veh). Rats receiving MP-10 (2.5 mg/kg) exhibited complete extinction of preference by the second and third extinction test (Mor/MP-10). The place preference score is defined as the difference in time spent in drug-paired compartments. Data are expressed as mean ± SEM. *P < 0.05, **P < 0.01 compared between Mor/Veh and Sal/Veh group. #P < 0.05 compared between Mor/MP-10 and Sal/Veh group. & P < 0.05 compared between Mor/Veh and Mor/MP-10. N = 6-7 per group.

Repeated measure two-way ANOVA of the data (Figure 3) reveals a significant difference in time (F_(4, 80)_ = 3.030, P < 0.05) and drug (F_(2, 80)_ = 13.64, P < 0.001), but not in time × drug interaction (F_(8, 80)_ = 0.9646, P > 0.05). Bonferroni post-test analysis revealed significant differences in the time spent in the morphine-paired compartment at the post-conditioning test (t = 2.734, P < 0.05), the first extinction test (t = 3.451, P < 0.01), and the second extinction test (t =3.226, P < 0.01) between Mor/Veh and Sal/Veh groups. Rats treated with 2.5 mg/kg MP-10 during extinction training exhibited significant differences only at the post-conditioning test (t = 2.793, P < 0.05) and the first extinction test (t = 2.782, P < 0.05). No preference was detected at the second and third extinction tests. At the second extinction test, a significant difference between Mor/Veh and Mor/MP-10 (t = 2.778, P < 0.05) groups in the time spent in the drug-paired compartment was obtained. The results suggest that 2.5 mg/kg MP-10 treatment facilitates extinction of morphine-acquired CPP.

### MP-10 causes long-lasting changes in expression of pCREB and ΔFosB immunoreactivity in morphine CPP tested rats

**Figure 4:**
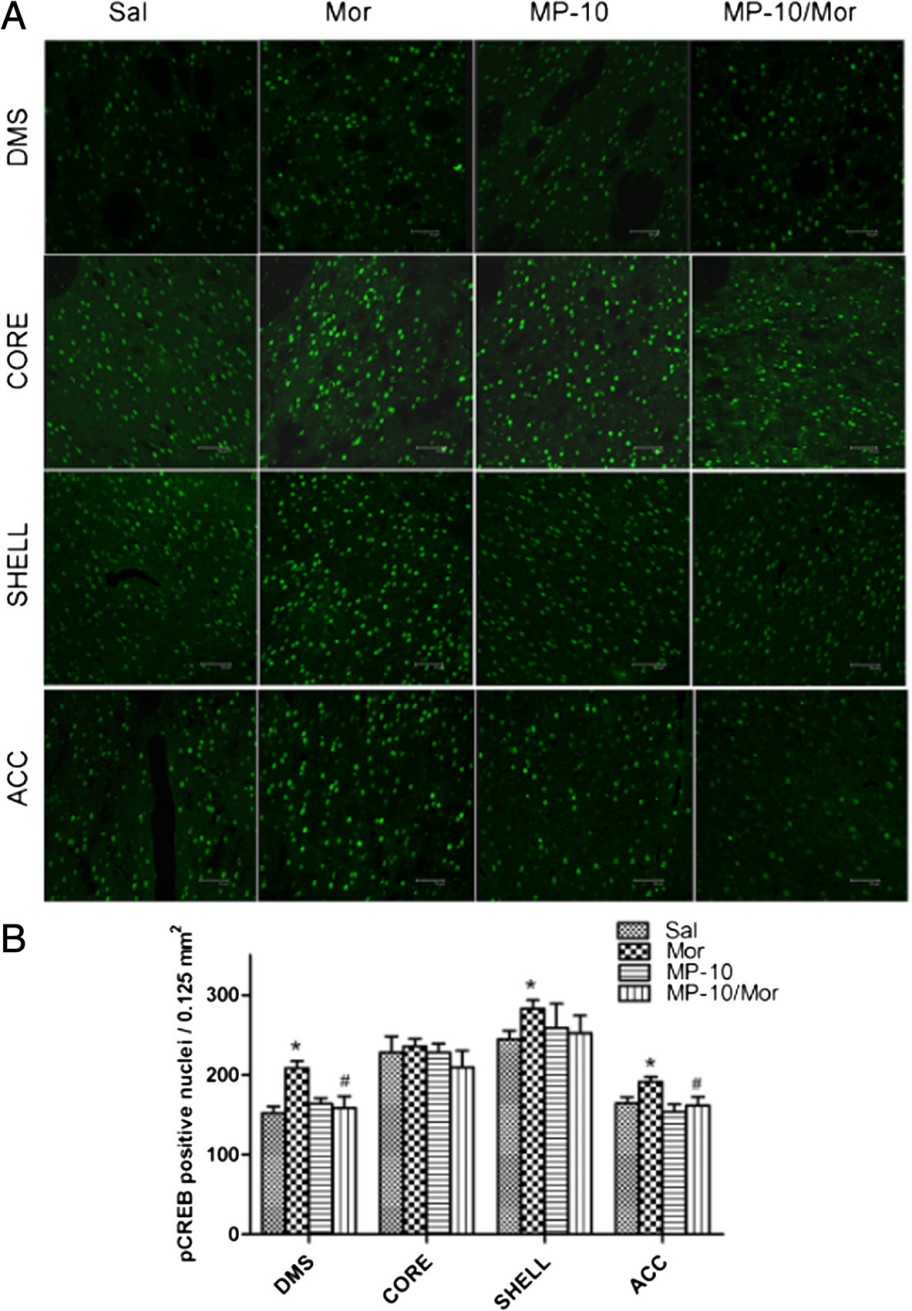
**MP-10 (2.5 mg/kg) causes long-lasting changes in expression of pCREB immunoreactivity. (A)** Immunofluorescence images of pCREB in different brain areas from rats receiving saline (Sal, 2 ml/kg, s.c.), morphine (Mor, 10 mg/kg, s.c.), MP-10 (MP-10, 2.5 mg/kg, s.c.) or a combination of both drugs (MP-10/Mor). MP-10 was administered 30 min prior to morphine and after CPP testing (scale bar represents 50 μm). **(B)** Quantification of pCREB positive nuclei. Data are expressed as mean ± SEM and analyzed by two-way ANOVA followed by Bonferroni posttests. *P < 0.05 when comparing between morphine and saline groups. #P < 0.05 when comparing the morphine group to the drug combination group. N = 5 per group. DMS: dorsomedial striatum; CORE: NAc core; SHELL: NAc shell; ACC: anterior cingulate cortex.

**Figure 5:**
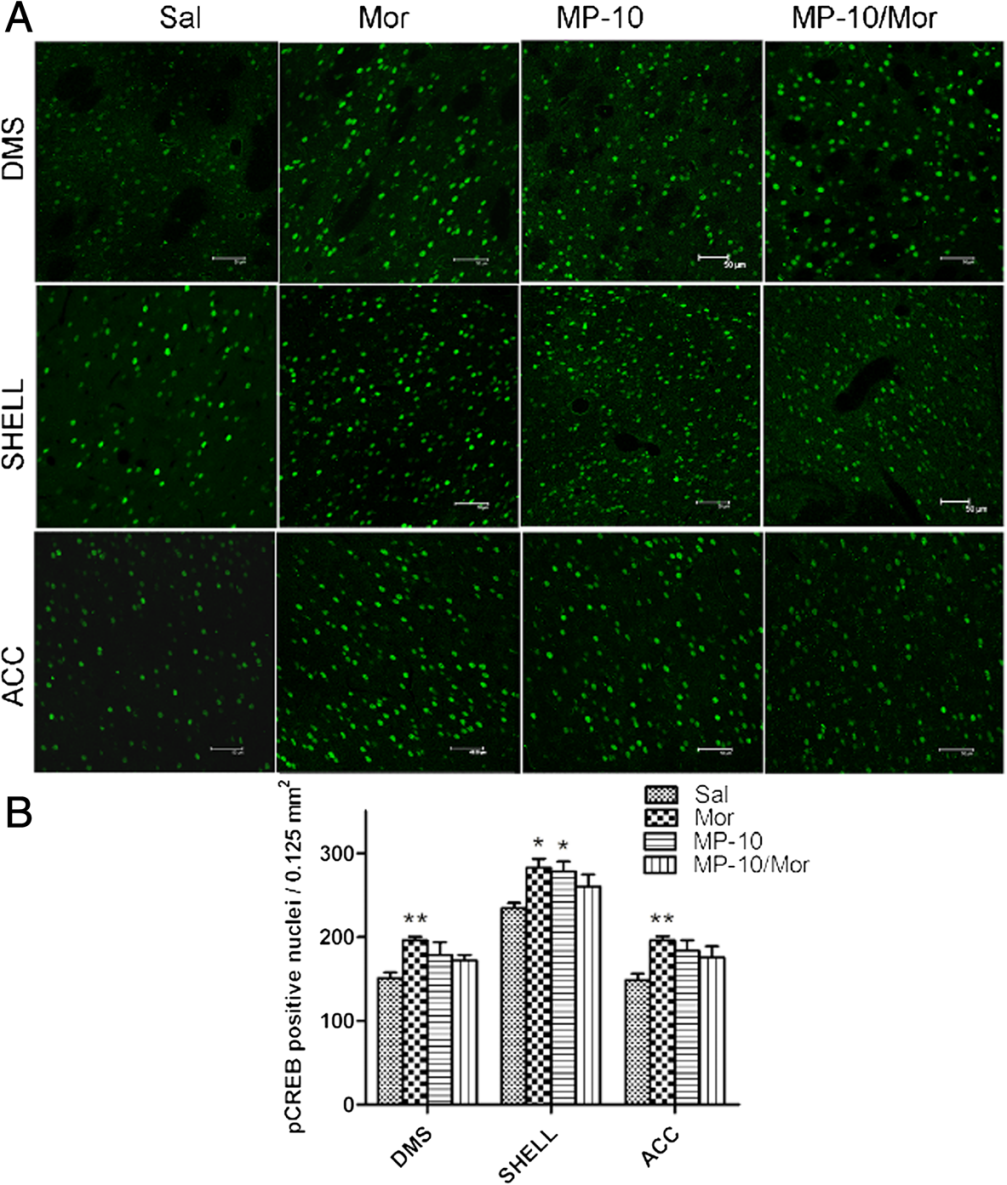
**MP-10 (10.0 mg/kg) causes long-lasting changes in expression of pCREB immunoreactivity. (A)** Immunofluorescence images of pCREB in different brain areas from rats receiving saline (Sal, 2 ml/kg, s.c.), morphine (Mor, 10 mg/kg, s.c.), MP-10 (MP-10, 10.0 mg/kg, s.c.) or a combination of both (MP-10/Mor, MP-10 was administared 30 min prior to morphine) after CPP testing (scale bar represents 50 μm). **(B)** Quantification of pCREB positive nuclei. Data are expressed as mean ± SEM and analyzed by two-way ANOVA followed by Bonferroni posttests. *P < 0.05, **P < 0.01 when compared with saline group. N = 5 per group. DMS: dorsomedial striatum; SHELL: NAc shell; ACC: anterior cingulate cortex.

CREB and ΔFosB are important transcriptional factors which are believed to play important roles in the development of CPP. Acute morphine and PDE inhibition, by altering the cAMP/cGMP content, were shown to increase the expression of pCREB [22,27] and ΔFosB [28]. We therefore, investigated the potential long-lasting effects of MP-10 on the expression of these transcription factors 48 h hours after acquisition of morphine CPP. As shown in Figures 4 and 5, two-way ANOVA analyses indicate that morphine significantly increased in the number of pCREB- immunoreactive cells in DMS, ACC, and NAc shell, but not in NAc core in rats that developed morphine CPP. Although MP-10 alone at the dose of 2.5 mg/kg did not produce a long-lasting increase in the number of pCREB-positive nuclei in any of the regions assessed, but significantly decreased morphine-induced pCREB expression in DMS (F_(1, 16)_ = 4.643, P < 0.05; t = 2.965, P < 0.05) and ACC (F_(1,16)_ = 4.548, P < 0.05; t = 2.634, P < 0.05). In contrast, 10.0 mg/kg MP-10 alone produced significant long-lasting increases in the number of pCREB- immunoreactive cells in NAc shell (F_(1,16)_ = 9.666, P < 0.01; t = 3.091, P < 0.05); but not in DMS, NAc shell, and ACC.

**Figure 6:**
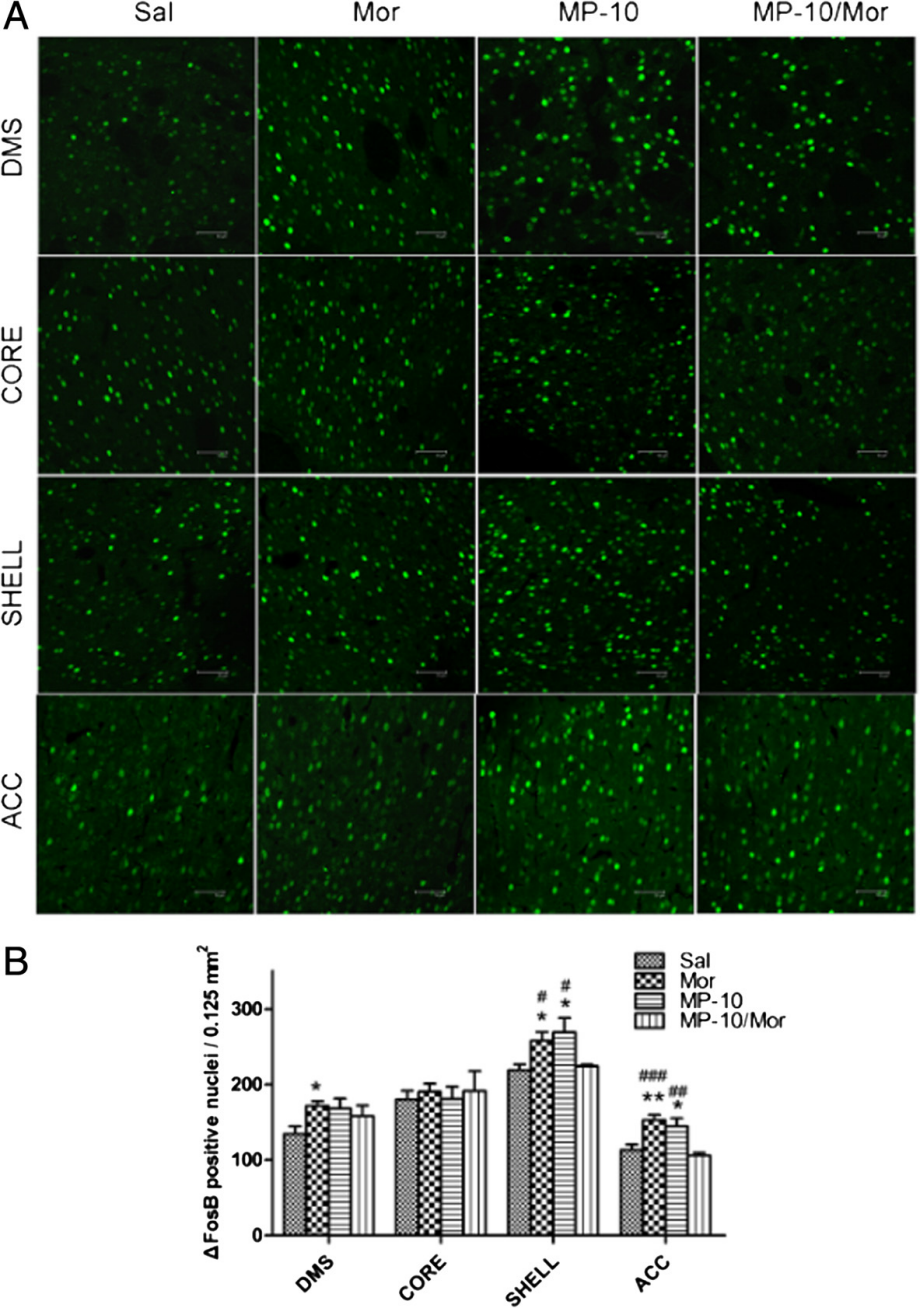
**MP-10 (2.5 mg/kg) causes long-lasting changes in expression of ΔFosB immunoreactivity. (A)** Immunofluorescence images of ΔFosB in different brain areas from rats that received saline (Sal, 2 ml/kg, s.c.), morphine (Mor, 10 mg/kg, s.c.), MP-10 (MP-10, 2.5 mg/kg, s.c.) or combination of both MP-10/Mor, (MP-10 was administration 30 min prior to morphine and after CPP testing (scale bar, 50 μm). **(B)** Quantification of ΔFosB-positive nuclei. Data are expressed as mean ± SEM and analyzed by two-way ANOVA followed by Bonferroni posttests. *P < 0.05, **P < 0.01 when compared with saline group. #P < 0.05, ##P < 0.01, ###P < 0.001 when compared with MP-10/Mor group. N = 5 per group. DMS: dorsomedial striatum; CORE: NAc core; SHELL: NAc shell; ACC: anterior cingulate cortex

**Figure 7:**
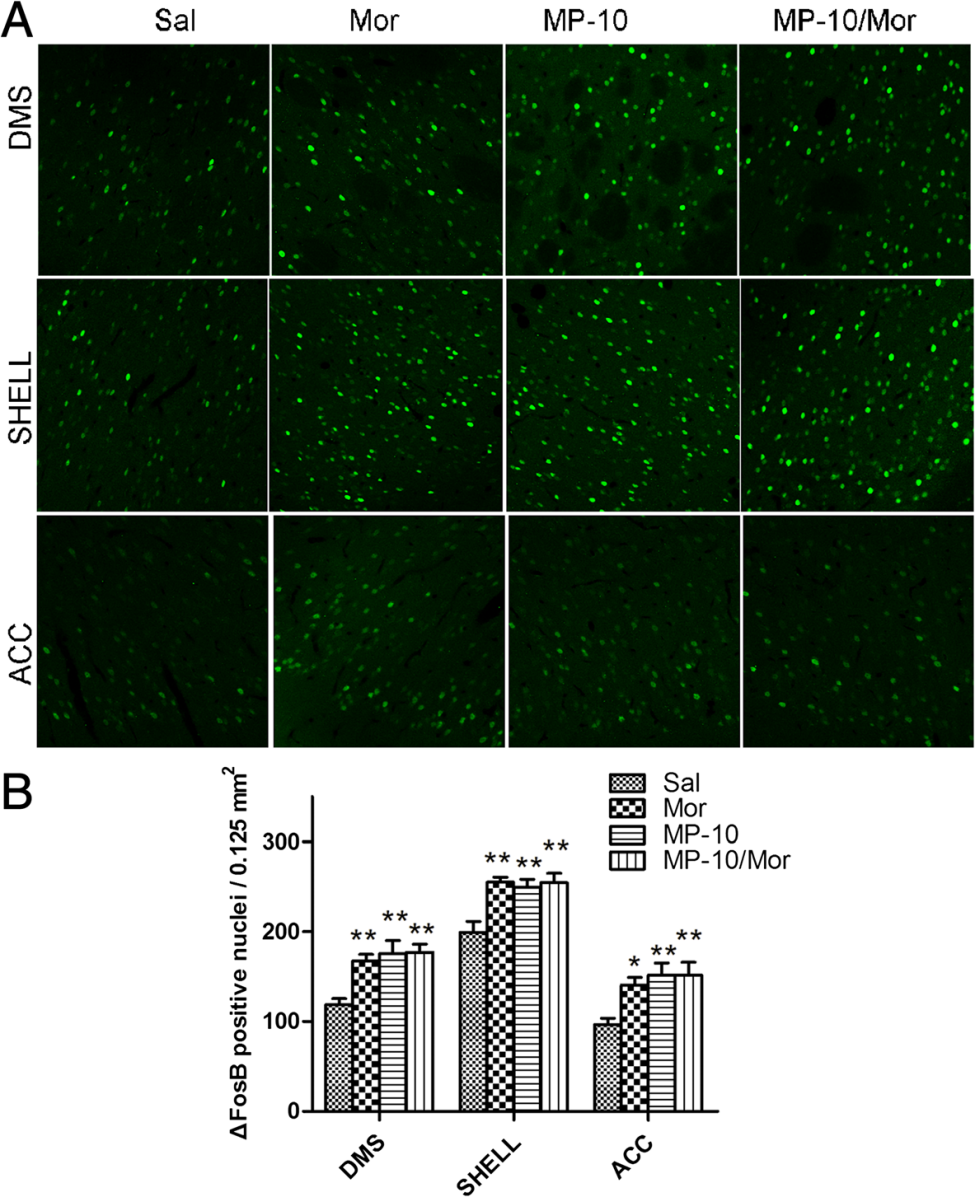
**MP-10 (10.0 mg/kg) causes long-lasting changes in expression of ΔFosB immunoreactivity. (A)** Immunofluorescence images of ΔFosB in different brain areas from rats that received saline (Sal, 2 ml/kg, s.c.), morphine (Mor, 10 mg/kg, s.c.), MP-10 (MP-10, 10.0 mg/kg, s.c.) or combination of both MP-10/Mor, (MP-10 was administration 30 min prior to morphine) after CPP testing (scale bar, 50 μm). **(B)** Quantification of ΔFosB-positive nuclei. Data are expressed as mean ± SEM and analyzed by two-way ANOVA followed by Bonferroni posttests. *P < 0.05, **P < 0.01, when compared with saline group. N = 5 per group. DMS: dorsomedial striatum; SHELL: NAc shell; ACC: anterior cingulate cortex.

ΔFosB is a splice variant of the FosB protein and belongs to the Fos family. Chronic administration of drugs of abuse, including morphine, induces long-lasting expression of ΔfosB that persists even after cessation of drug treatment [23]. In agreement with previous observation, we detected (Figures 6 and 7) that morphine produced significant increases in the number of ΔFosB- immunoreactive cells in DMS, NAc shell, and ACC. MP-10 alone, either at 2.5 mg/kg or 10.0 mg/kg, increased the number of ΔFosB- positive nuclei in NAc shell (F_(1, 16)_ = 11.24, P < 0.01; t = 2.787, P < 0.05 and F_(1, 16)_ = 10.23, P < 0.01; t = 4.143, P < 0.01) and ACC (F_(1, 16)_ = 26.20, P < 0.001; t = 2.869, P < 0.05 and F_(1, 16)_ = 8.606, P < 0.01; t = 2.749, P < 0.05). However, inhibition of PDE10A by MP-10 (2.5 mg/kg) significantly decreased morphine-induced expression of ΔFosB in ACC (t = 4.370, P < 0.001) and NAc shell (t = 2.477, P < 0.05). In contrast, 10 mg/kg MP-10 enhanced morphine-induced expression of ΔFosB in NAc shell (t = 3.710, P < 0.01), ACC (t = 3.455, P < 0.01), and DMS (t = 4.097, P < 0.01).

## Discussion

The cAMP signaling cascade plays a critical role in the development and maintenance of addiction to drugs of abuse [29]. Modulation of cAMP content by altering its production or hydrolysis has become an important focus in developing potential therapeutic approaches for addiction. For instance, a D1 receptor antagonist, which blocks D1 receptor-mediated stimulation of cAMP, or a D2 receptor antagonist, which abolishes D2 receptor-mediated inhibition of cAMP formation, was shown to have some potential therapeutic effects in drug abuse [30,31]. PDE is a large family of enzymes that play a key role in modulation of cAMP and/or cGMP cellular content via their ability to hydrolyze the two second messengers. It was shown that administration of the PDE4 inhibitor rolipram [18,32] and the PDE9 inhibitor BAY-73-6691 [33] suppressed both the acquisition of cocaine CPP and its extinction. Unlike the wide distribution of PDE4 in brain, PDE10A is selectively expressed in medium spiny neurons (MSNs) of striatum. The present study provides evidence that selective inhibition of PDE10A significantly attenuates the acquisition of, and facilitates the extinction of morphine-induced CPP. This suggests that PDE10A-mediated breakdown of cyclic nucleotide in striatal neurons may participate in the reward pathway of addictive drugs. Our results thus provided the first evidence for the potential therapeutic effect of a PDE10A inhibitor in morphine addiction. It should be noted that acute inhibition of PDE10 failed to alter the expression of morphine-induced CPP, suggesting that the neuroplasticity induced by repeated MP-10 treatment may underlie drug-attenuated acquisition, and drug-facilitated extinction.

MP-10 specifically inhibited PDE10A of MSN in striatum and increased cAMP concentration in these neurons, an effect that functionally resembles D1 receptor stimulation or D2 receptor inhibition with regard to the production of cAMP. It is well known that almost all drugs of abuse exert their rewarding effect through the release of dopamine and activation of dopamine receptors in nucleus accumbens (NAc) [16]. Systemic administration of D2 receptor antagonists have been shown to reverse morphine-induced CPP [31,34]. Those observations are in line with the effect of 2.5 mg/kg MP-10 on morphine-induced CPP in the current study. It is reasonable to assume that this effects may be attributed to the increase in cAMP content in striatum by MP-10, as it has been shown before that 3 mg/kg of MP-10 significantly elevated striatal cAMP and cGMP [27]. It should be noted that higher doses of MP-10 (5, 10 mg/kg) failed to inhibit morphine CPP. One possible explanation for this maybe that an optimal intracellular concentration of cAMP/cGMP is required and that higher levels result in damaged neuronal functions. In support, we found that MP-10 (10 mg/kg) enhanced morphine-induced expression of ΔFosB in NAc shell, ACC, and DMS. Indeed, we found that both 5 and 10 mg/kg of MP-10 produced catalepsy as tested with a vertical wire-mesh grid, in which the descent latency of 5, 10 mg/kg of MP-10 is significantly more than that of 2.5 mg/kg (data not shown). Another possible cause maybe attributed to the transit and weak CPP (Figure 2) observed when a high dose of MP-10 is administered alone.

To further understand the neurobiological mechanisms underlying the effect of PDE10A inhibition on morphine- induced CPP, we checked the expression of pCREB and ΔFosB in nucleus accumbens (NAc), dorsomedial striatum (DMS), and anterior cingulate cortex (ACC). In agreement with previous reports [25,35], we detected elevations of pCREB and ΔFosB in rat brain regions of morphine CPP animals 48 hours after the last drug administration. MP-10 at the dose of 2.5 mg/kg significantly decreased morphine-induced pCREB expressions in DMS and ACC as well as the expression of ΔFosB in NAc shell and ACC. This may be a result of negative feedback of striatal neurons onto midbrain cells. There are a minority of MSNs which make up the so-called “patch” population and target only dopaminergic cells in the midbrain, ventral tegmental area (VTA) and substantia nigra pars compacta (SNc) [9,36]. MP-10 targeted and activated striatal MSNs including those projecting to the midbrain and the increased release of GABA from those MSNs may have altered the disinhibition caused by activation of mu-opioid receptors in VTA [9], thus, abolishing morphine-induced dopamine release in striatum and PFC [16]. This is supported by the observation that MP-10 blocks D-amphetamine-induced dopamine efflux in NAc through D1-regulated feedback control of midbrain dopamine neurons [37]. As a result, morphine-induced increases of pCREB in ACC and of ΔFosB in NAc shell and ACC were thus suppressed by MP-10. Ultimately and importantly, morphine-induced CPP was also inhibited. In contrast to the effect of 2.5 mg/kg MP-10, administration of 10 mg/kg of the drug enhanced morphine-induced pCREB and ΔFosB in DMS, NAc shell, and ACC. This may provide a potential explanation for why 10.0 mg/kg MP-10 did not inhibit the acquisition of morphine CPP. Since it is known that ΔFosB plays an essential role in mediating a state of prolonged sensitization to addictive drugs this may underlie the increased drive and motivation for drug seeking behavior [25]. Therefore, MP-10 (2.5 mg/kg) attenuated morphine-increased ΔFosB levels in ACC and shell of NAc may provide a potential molecular mechanism for the anti-relapse effect of the drug. Given the fact that both morphine and MP-10 alone increased the expression of ΔFosB, it will be of great interest to investigate how MP-10 inhibits the expression of ΔFosB in those brain regions.

We did not detect any significant changes in expression of either pCREB or ΔFosB in NAc core among the experimental groups. Interestingly, a previous study showed that nicotine dependent conditioning resulted in elevated pCREB level in the NAc shell but not in NAc core in mice [38]; and cocaine CPP was accompanied by significant increases in expression of Fos in the shell rather than the core of NAc [39]. In addition, it was reported that decreased pCREB expression was observed in palladium of rats withdrawing from morphine-induced behavioral sensitization [40]. The reason for this discrepancy may be associated with the different paradigms employed and/or the distinct functional involvement of different brain regions in response to drug of abuse.

## Conclusion

In conclusion, we found that MP-10 administration at the dose of 2.5 mg/kg suppressed the acquisition of morphine-induced CPP through inhibiting morphine- induced increases in pCREB and ΔFosB in brain regions within the rewarding circuit, such as dorsomedial striatum, shell of nucleus accumbens, and anterior cingutate cortex. Our results reveal that MP-10 can inhibit morphine -induced CPP and thus it may have therapeutic potential in opioid abuse.

## Methods

### Animals and drugs

#### Animals

Male Sprague–Dawley rats, weighed 150–200 g, were purchased from Shanghai Laboratory Animal Co. LTD (Shanghai, China). The rats were habituated for one week prior to the experiments. All animals were housed in constant temperature (21 ± 2°C) and humidity (about 60%) with a 12 h light/dark cycle. Food and water were available *ad libitum*. All experimental protocols were approved by the Institutional Animal Care and Use Committee of Soochow University and were conducted in accordance with the U.S. National Institutes of Health Guide for the Care and Use of Laboratory Animals.

#### Drugs

MP-10 (2-[4-(1-methyl-4-pyridin-4-yl-1H-pyrazol-3-yl)-phenoxymethyl]-quinoline succinic acid), purchased from Shanghai Pharmaresource Inc, was dissolved in 5% 2-hydroxypropyl-β-cyclodextrine/0.5% carboxymethylcellulose sodium for subcutaneous injection (s.c.). Morphine hydrochloride was purchased from Shenyang First Pharmaceutical Factory, Northeast Pharmaceutical Group Corp. (Shenyang, China) and dissolved in saline. Controls were injected (s.c.) with the same volume of either 5% 2-hydroxypropyl-β -cyclodextrine/0.5% carboxymethylcellulose sodium or saline. All drugs or vehicles were administered with a volume of 2 ml/kg.

### Behavioral procedures

#### Apparatus

The apparatus for CPP conditioning and testing consisted of eight identical plexiglas/polyvinyl chloride (PVC) boxes purchased from Jiliang Ltd. (Shanghai, China). The boxes were composed of two compartments with distinct visual and textural cues. One compartment had white walls and a fine wire mesh floor (0.5 × 0.5 cm^2^), whereas another had black walls and a wide grid floor with metal rods spaced 1.6 cm apart. These two compartments were separated by a removable wall with an arched gateway to allow the free movement of animals through the whole apparatus during the testing session, whereas during the conditioning session, the separating wall was closed to restrict the animal in their designated conditioning compartment. The movements within the compartments and time spent in each compartment were recorded and automatically measured by a computer through interruption of infrared beams by the test animals.

#### Conditioned place preference (CPP)

Animals were habituated in the test room for 1 hr before the experiment began. The procedures were previously described and used with minor modification [1,4-6]. The 10-day CPP procedure included three main phases: a pre-conditioning test session, eight daily conditioning sessions and a post-conditioning test session. On day 0, the base-line preferences of animals were determined by placing them for 15 minutes in the apparatus with the separating wall and the arched gateway present (pre-conditioning test). Rats that spent more than 70% of the session time in one compartment were excluded because of their strong unconditioned preference. During the second phase, conditioning was performed using an unbiased, balanced protocol, after the separating wall was inserted to restrict movement of the animal. The order of injection (drug or vehicle), and the compartment paired with the drug or saline, was counterbalanced within each group. In this phase, each rat was trained for eight consecutive days with alternative injections of morphine (10 mg/kg, s.c.) and saline (2 ml/kg, s.c.). On days 1, 3, 5, and 7, rats were injected with morphine and immediately confined to the drug-paired compartment for 45 min before returning to their home cages. On day 2, 4, 6, and 8, rats were injected with saline and confined for 45 min in the opposite compartment (saline-paired compartment). On the third phase, day 9, the separating wall was reversed again with the arched gateway opening to allow rats to freely explore for 15 min. The time spent in each compartment during this session was recorded (post-conditioning test). The CPP score was defined as the difference of the time spent in the drug-paired compartment during the pre- and post-conditioning tests.

### Experimental protocols

To study the effect of MP-10 on the acquisition of morphine-induced CPP, the same general procedure as described above was used to develop morphine CPP, except for the following modifications: MP-10 (1.25-10 mg/kg, s.c.) or vehicle was administered 30 min before each morphine or saline conditioning sessions. On day 9, the animals were tested for morphine CPP in a drug free state. An additional experiment was carried out to evaluate whether MP-10 alone could induce CPP: rats received MP-10 (2.5-10 mg/kg, s.c.) instead of morphine following the above-mentioned conditioning procedure. In this experiment, administration of 10.0 mg/kg MP-10 caused a mild preference trend, so a second test was repeated one week afterward.

To observe the effect of MP-10 on the expression of established morphine CPP, morphine (10 mg/kg, s.c.) was used during the conditioning sessions in all the groups except the saline-treated group. On the test day, vehicle (2 ml/kg, s.c.) or MP-10 (2.5 mg/kg, s.c.) was injected 30 min prior to placement in the apparatus, with free access to the two compartments for 15 min.

The effect of MP-10 (2.5 mg/kg) on extinction to morphine CPP was determined in a separate set of animals. The general procedure described above was used to establish morphine CPP. After testing for the expression of CPP in a drug-free state on the day 9, extinction conditioning sessions were initiated. This extinction procedure is very similar to the original acquisition training. On day 10, 12, 14, 16, MP-10 (2.5 mg/kg, test group) or vehicle (2 ml/kg, control group) was administered prior to restricting the animals to the same drug-paired compartment as in acquisition training, whereas on day 11, 13, 15, 17, vehicle was administered prior to exposure to the saline-paired compartment before placement in the home cage. This extinction procedure was repeated three times (Days 10–17, 19–26, and 28–35). During this extinction procedure, rats in the saline group received daily vehicle (2 ml/kg) injections prior to alternating drug-paired or saline-paired compartment placement. On day 18, 27, and 36, the place preference was tested by allowing rats free access for 15 min. The place preference score is defined as the time spent in drug-paired compartments. Extinction of place preference occurred when there was no significant difference in time spent in drug-paired and saline-paired chambers among groups that were previously exhibited morphine CPP.

### Immunofluorescence Procedure

#### Tissue preparations

In the experiment of the effect of MP-10 (2.5 mg/kg and 10.0 mg/kg) on the acquisition of morphine-induced CPP, immediately after the post-conditioning test had finished, rats (n = 5 animals per group) were deeply anesthetized with sodium pentobarbital (100 mg/kg, i.p.) and perfused transcardially with saline, followed by 4% paraformaldehyde in 0.1 M phosphate-buffered saline (PBS, pH 7.4). The brains were then removed and post-fixed overnight at 4°C using the same fixative solution. Brain tissues were transferred to 15% sucrose for 24 h before rinsing in 30% sucrose for an additional 24 h at 4°C. As shown in Figure 1, coronal sections (20 μm) were prepared with a freezing microtome following the rat brain atlas of Paxinos and Watson (2004, 5th ed.) as follows: anterior cingulate cortex (bregma +2.16), dorsomedial striatum (bregma +1.56), NAc (bregma +1.56).

#### Protein immunofluorescence

For immuno-staining, tissue sections were first washed in 0.01 M PBS (3 × 10 min) and then incubated in 0.01 M PBS containing 10% normal goat serum (NGS), 0.1% Triton X-100, and 3% bovine serum albumin for 1 h to decrease non-specific staining. The sections were then incubated for 24 h at 4°C in 0.01 M PBS containing anti-ΔFosB (SC-48; 1:400; Santa Cruz) or anti-pCREB rabbit polyclonal antibody (06–519; 1:400; Millipore). Unbound primary antibodies were washed in 0.01 M PBS (3 × 10 min) prior to 24 h incubation at 4°C with FITC-conjugated goat anti-rabbit secondary antibody (1:400; Sigma-Aldrich) followed rinse in PBS for 3 × 10 min. Finally, 4′, 6′-diamidino-2-phenylindole (DAPI) was added to label nuclei, and the sections were mounted with mounting medium for fluorescence observation.

#### Image analysis

Quantification of ΔFosB and pCREB immuno-reactivity was conducted using a Leica TCS-SP2 (Leica Instruments, Germany) laser confocal microscope set at 40× magnification and counted by an observer blind to treatment conditions with the Image-Pro Plus software. For all regions, a size of 0.125 mm^2^ area was counted for each section in each hemisphere. Thus there were a total of six sample areas which were counted for each animal (i.e., 1 sample area/2 hemispheres/3 sections). The counts from all six sample areas from a particular region were averaged to obtain a mean number of immunoreactive cells/0.125 mm^2^.

### Statistical analysis

Data were expressed as mean ± SEM and were analyzed by Graph Pad Prism® (Version 5.0) software. The results from the acquisition and expression of the CPP test were analyzed using one-way ANOVA followed by the Bonferroni’s multiple comparison tests. The data from immunofluorescence experiments were analyzed with two-way ANOVA followed by Bonferroni post-tests. Repeated measurement of two-way ANOVA was conducted followed by Bonferroni post-test to assess the differences of CPP scores in extinction of CPP. Values of p < 0.05 were considered statistically significant.

## Abbreviations

ACC: Anterior cingulate cortex
cAMP: Cyclic adenosine monophosphate
CPP: Conditioned place preference
CREB: cAMP response element binding protein
D1 receptor: Dopamine type 1 receptor
D2 receptor: Dopamine type 2 receptor
DMS: Dorsomedial striatum
GABA: Gamma aminobutyric acid
MSNs: Medium spiny neurons
NAc: Nucleus accumbens
PDE: Phosphodiesterase
SNc: Substantia nigra pars compacta
VTA: Ventral tegmental area
